# Lean diabetes in middle-aged adults: A joint analysis of the German DIVE and DPV registries

**DOI:** 10.1371/journal.pone.0183235

**Published:** 2017-08-21

**Authors:** Bettina Hartmann, Stefanie Lanzinger, Peter Bramlage, Felix Groß, Thomas Danne, Siegfried Wagner, Dietmar Krakow, Artur Zimmermann, Christian Malcharzik, Reinhard W. Holl

**Affiliations:** 1 Department of Gastroenterology and Diabetology, Klinikum Ludwigshafen, Ludwigshafen, Germany; 2 Institute of Epidemiology and Medical Biometry, ZIBMT, University of Ulm, Ulm, Germany; 3 German Center for Diabetes Research (DZD), Neuherberg, Germany; 4 Institute for Pharmacology and Preventive Medicine, Mahlow, Germany; 5 Praxis Dr. Groß, Murnau, Germany; 6 Diabeteszentrum für Kinder und Jugendliche, Kinderkrankenhaus auf der Bult, Hannover, Germany; 7 Department of Internal Medicine II, DONAUISAR Klinikum, Deggendorf, Germany; 8 Diabeteszentrum Forchheim, Forchheim, Germany; 9 Praxis Dr. Zimmermann- Diabeteszentrum Bad Aibling, Bad Aibling, Germany; 10 Diabetes Kröpcke, Hannover, Germany; Shanghai Diabetes Institute, CHINA

## Abstract

**Aims:**

To assess differences in demographics, treatment and outcome of lean (LD) compared to overweight and obese people with diabetes clinically classified as type 2 diabetes mellitus (T2DM).

**Materials and methods:**

We combined data from the German DIVE (Diabetes Versorgungs-Evaluation) and DPV (Diabetes-Patienten-Verlaufsdokumentation) databases to produce a large cohort of people with T2DM. The characteristics of people with Body Mass Index (BMI) <25 kg/m^2^, ≥25–30 kg/m^2^ and ≥30 kg/m^2^ aged 30 to 50 years were compared, including demographics, cardiovascular (CV) risk factors, comorbidities and outcomes.

**Results:**

A total of 37,870 people were included in the analysis, 3,191 of these (8.4%) had a BMI < 25 kg/m^2^. LD reported more nicotine (41.6% of 2,070 vs. 38.1% of 6,070 and 33.4% of 16,823; P<0.001)and alcohol consumption (12.0% of 1,282, 10.3% of 3,594 and 6.6% of 9,418; P<0.001)compared to overweight and obese people. More LD were treated with insulin in comparison to the other subgroups (short acting insulin 33.1% of 3,191 vs. 28.4% of 9,234 and 28.0% of 25,445; P <0.001; long acting insulin 31.3% of 3,191 vs. 28.9% of 9,234 and 29.3% of 25,445; P = 0.043). Regression models adjusted for age, gender and diabetes duration showed a 2.50 times higher odds ratio (OR) for hypoglycemia and a 2.52 higher OR for mortality in LD compared to the BMI subgroup ≥30 kg/m^2^.

**Conclusions:**

LD is associated with an increased risk of hypoglycaemia and death. Patients are characterized by male gender, lifestyle habits as smoking and alcohol consumption while cardiovascular comorbidities are less important. In comparison to patients of the other weight groups they are treated with insulin more often and considerably less with metformin.

## Introduction

The majority of patients with type 2 diabetes mellitus (T2DM) are obese or overweight. Few studies have investigated the subgroup of patients with a clinical diagnosis of type 2 diabetes and a low to normal body mass index (BMI) (< 25 kg/m^2^). In previous studies, percentages of these lean diabetic participants ranged from 7.5% to 21% [1–5]. In these studies, participants with lean diabetes were mainly males and had higher frequency of insulin use indicating rapid beta cell failure. Lean diabetes patients might have higher total and non- cardiovascular mortality when compared to obese diabetic patients [1, 6], whereas, most studies reported a lower cardiovascular mortality compared to participants with BMI > 25 kg/m^2^ [2, 3]. According to available diagnostic tools in everyday clinical practice, the subgroup of non-obese T2DM patients and obese T2DM patients share the same phenotype of diabetes but are different in terms of etiology and pathophysiology. In previous studies, participants with T1DM, latent onset autoimmune diabetes of the adult (LADA), maturity onset diabetes of the young (MODY, especially type 3), participants with secondary diabetes as a consequence of pancreatitis, participants with type 2 diabetes and wasting diseases (i.e. malignancies, tuberculosis, aquired immunodeficiency syndrome) as well as T2DM with a low to normal body weight were included in the subgroup of lean diabetes, depending on the respective inclusion criteria [7, 8].

The aim of our analysis was to characterize participants with the phenotype of lean type 2 diabetes mellitus (T2DM), their diabetes therapies and outcomes compared to overweight and obese participants in the DIVE and DPV databases consisting of participants from Germany.

## Materials and methods

### Study design and data sources

Data were obtained from the DIVE (Diabetes Versorgungs-Evaluation) registry and the DPV (Diabetes-Patienten-Verlaufsdokumentation) database. The DIVE registry was established in Germany in 2011 [9–13]. Patients with diabetes mellitus, regardless of their disease stage and treatment strategy, were enrolled consecutively at 159 outpatient clinics across the country. Data were entered into local database using the DPV (Ulm University) or DIAMAX (Axaris software & systeme GmbH) software. All patients included in the DIVE registry provided written informed consent. The study protocol was approved by the ethics committee of the Medical School of Hannover. The DPV database was established in Germany in 1995 [14–16]. Approximately 450 centers in Germany currently use the DPV software. Every 6 months, locally documented data are anonymized and sent to the University of Ulm. The DPV initiative was approved by the ethics committee of the University of Ulm, and data collection was approved by local review boards.

Individuals with a clinical diagnosis of T2DM and aged 30 to 50 years, documented in DPV until September 2016 and in DIVE until December 2016 were included in the present analysis. Participants who tested positive for beta- cell autoantibodies were excluded (GAD-Antibodies, ICA, IA-2 antibodies and ZnT8 antibodies). Moreover, patients with low body weight as a consequence of wasting diseases such as malignancies, tuberculosis, alcoholism or chronic pancreatitis were also excluded from the DPV dataset. Because of technical reasons this was not possible for centers with the DIAMAX software.

### Documentation

Data regarding age, gender, BMI, blood pressure, CV risk factors (hypertension, hypercholesterolemia), lifestyle factors (smoking, alcohol consumption over 20 g per day, sedentary lifestyle), CV history, history of comorbidities and current comorbidities (during the most recent treatment year) were collected. In addition, details of diabetes therapy were documented. Hypercholesterolemia was defined as total cholesterol ≥ 200 mg/dl, LDL-cholesterol ≥ 160 mg/dl, HDL-cholesterol <40 mg/dl, triglycerides ≥ 150 mg/dl or medication with lipid lowering drugs. Coronary heart disease (CHD) was defined as a previous myocardial infarction or history of angina pectoris, hypertension was defined as blood pressure levels >140 mm Hg systolic or 90 mm Hg diastolic and/or receiving antihypertensive drugs, and being physically active less than once a week was considered as a sedentary lifestyle.

### Statistics

Categorical variables are presented as percentages. Continuous variables are presented as means with standard deviations (s.d.). Unadjusted comparisons were conducted using chi-squared test for binary and Kruskal-Wallis test for continuous variables. Logistic regression was performed to evaluate odds ratios for being a lean patient with type-2 diabetes and for CV risk factors, history of comorbidities, current comorbidities and death, respectively. Number of deaths may be underreported due to documentation based on diabetes practices. Data are given as unadjusted odds ratios (ORs), and adjusted for age, sex and diabetes duration. A two-sided P- value < 0.05 was considered statistically significant. Statistical analysis was performed using SAS version 9.4.

## Results

### Study population

**Fig 1:**
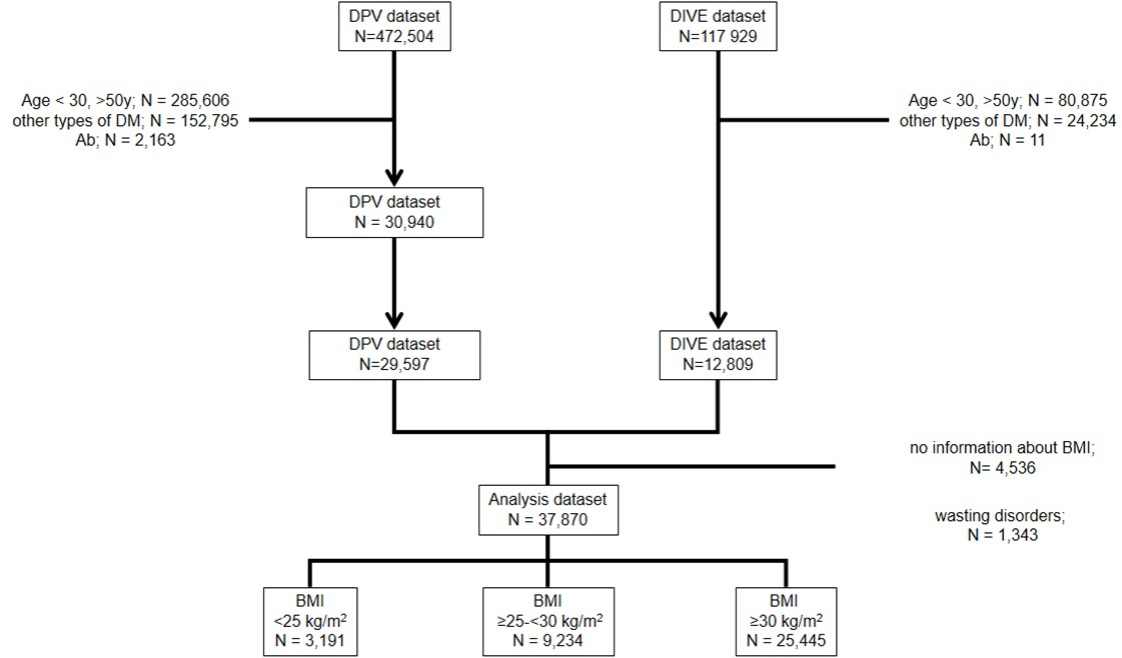
Patient flow. DM: diabetes mellitus; Ab: beta-cell autoantibodies; wasting disorders: malignancies, tuberculosis, chronic pancreatitis, alcoholism; BMI: body mass index.

A total of 37,870 participants (42.5% women) were included in the present analysis (Fig 1); 12,809 datasets were derived from the DIVE registry and 29,597 from the DPV initiative, 4,536 patients with missing BMI were excluded. The mean (s.d., minimum-maximum) age was 44.1 (5.42, predefined ranges 30–50) years, and the mean (s.d.) BMI was 34.1 kg/m^2^ (7.7). The mean (s.d.) duration of diabetes was 3.2 years (11.6). CV risk factors were highly prevalent, in particular obesity (49.8%), hypertension (59.9%), dyslipidemia (78.6%) and a sedentary lifestyle (85.1%).

A total of 3,191 (8.4%) participants (40.8% women) were assigned to the lean subgroup of participants with BMI <25 kg/m^2^, 9,234 (31.7% women) to the subgroup with BMI ≥25- <30 kg/m^2^ and 25,445 (44.4% women) to the subgroup with BMI ≥30 kg/m^2^ (Table 1), There were more male participants in the lean subgroup compared to the subgroup with BMI ≥ 30 kg/m^2^ (60.0% vs. 55.6%). CV risk factors were less prevalent in the lean BMI subgroup: hypertension in 35% and hypercholesterolemia in 78.0%. Concerning lifestyle, there were significantly more smokers (41.6% vs. 38.1% and 33.4%, P<0.001) and participants with alcohol consumption over 20 g per day in the lean BMI subgroup (12.0% vs. 10.3% and 6.6%, P<0.001) while sedentary lifestyle was equally distributed between the subgroups (78.0% vs. 77.0% and 78.8%; P = 0.185). Macrovascular comorbidities as acute coronary syndrome (ACS), history of myocardial infarction and history of peripheral artery disease (PAD) (ACS: 2.9% vs. 3.9% and 3.7% (P = 0.045), MI: 2.2% vs. 3.3% and 2.9% (P = 0.002), PAD: 6.1% vs. 5.9% and 6.7% (P = 0.046) were reported less frequently in the lean BMI subgroup while history of stroke was equally distributed in all subgroups. However, chronic kidney disease (CKD) as a manifestation of microvascular comorbidity was more prevalent in the lean BMI subgroup (7.6% vs. 5.6% and 7.0%, P< 0.001).

**Table 1:** Patient characteristics and multivariable adjusted “predictors” for participants with lean type 2 diabetes (BMI<25 kg/m^2^) vs. participants with BMI ≥30 kg/m^2^.

	Total	BMI		BMI		BMI		Unadjusted	Adjusted
		<25 kg/m^2^		≥25-<30 kg/m^2^		≥30kg/m^2^		BMI	BMI
								<25 vs. ≥30	<25 vs. ≥30
								kg/m^2^	kg/m^2^
								OR (95%CI)	OR (95%CI)^a^
	N	N		N		N			
Age (years, s.d.)	37870	3191	44.6	9234	44.8	25445	44.4	1.00	1.01
			(5.0)		(4.8)		(5.1)	(1.00; 1.02)	(1.00; 1.02)
Male (%)	37870	3191	60.0	9234	68.3	25445	55.6	1.19	1.19
								(1.11; 1.29)	(1.10; 1.29)
Systolic Blood Pressure	35410	2969	125.0	8655	130.3	23786	135.2	0.96	0.96
(mmHg, s.d.)			(16.5)		(16.5)		(16.8)	(0.95; 0.96)	(0.95; 0.96)
Diastolic Blood Pressure	35340	2966	77.5	8642	80.8	23732	83.2	0.9	0.95
(mmHg, s.d.)			(10.2)		(10.3)		(10.6)	(0.94; 0.95)	(0.94; 0.95)
**Lifestyle factors**									
Current smoker (%)	24963	2070	41.6	6070	38.1	16823	33.4	1.42	1.41
								(1.30; 1.56)	(1.28; 1.55)
Alcohol consumption (%)^b^	14294	1282	12.0	3594	10.3	9418	6.6	1.93	1.76
								(1.60; 2.33)	(1.46; 2.14)
Sedentary lifestyle (%)	10720	903	78.0	2626	77.0	7191	78.8	0.95	0.94
								(0.80; 1.12)	(0.80; 1.12)
**Comorbidities**									
ACS (%)	37870	3191	2.9	9234	3.9	25445	3.7	0.78	0.73
								(0.62; 0.96)	(0.58; 0.91)
Hypertension (%)	35767	2988	35.0	8717	50.0	24062	64.9	0.29	0.27
								(0.27; 0.32)	(0.25; 0.30)
CKD (%)	30,782	2567	7.6	7537	5.6	20678	7.0	1.09	1.13
								(0.93; 1.28)	(0.96; 1.32)
Hypercholesterolemia (%)	28308	2305	78.0	6950	87.7	19053	89.7	0.41	0.38
								(0.37; 0.46)	(0.34; 0.43)
History of stroke (%)^c^	37870	3191	1.7	9234	1.5	25445	1.6	1.09	1.10
								(0.82; 1.45)	(0.82; 1.48)
History of MI (%)	37870	3191	2.2	9234	3.3	25445	2.9	0.75	0.71
								(0.59; 0.97)	(0.55; 0.91)
History of PAD (%)	37870	3191	6.1	9234	5.9	25445	6.7	0.90)	0.88
								(0.77; 1.05	(0.75; 1.04)

N, data available for analysis; BMI, body mass index; ACS, acute coronary syndrome; CKD, chronic kidney disease; MI, myocardial infarction; PAD, peripheral artery disease; s.d., standard deviation; OR, odds ratio; CI, confidence interval.

^a^ adjusted for age, sex and diabetes duration

^b^ defined as more than 20g alcohol consumption per day

^c^ Includes ischaemic and haemorrhagic stroke.

### Laboratory values

HbA1c differed statisticly significant with 8.03% in the lean body weight group, 7.99% and 8.02% in the overweight and obese subgroup. Fasting blood glucose was significantly higher with increasing BMI subgroup. However, both differences in parameters of glucose metabolism seem to be clinically insignificant. Lipid profile was more favorable in the lean body mass group (Table 2).

**Table 2:** Laboratory values (last recorded profile).

	Total	BMI		BMI		BMI		P- Value
	N	<25 kg/m^2^		≥25-<30 kg/m^2^		≥30 kg/m^2^		
		N		N		N		
HbA1c (%)	35585	2968	8.03	8646	7.99	23971	8.02	<0.001
			(2.5)		(2.3)		(2.1)	
Fasting	23629	1985	166.6	5814	167.2	15830	168.5	<0.001
blood glucose			(101.2)		(98.5)		(81.8)	
LDL cholesterol	23734	1957	119.7	5759	125.0	16018	122.4	<0.001
(mg/dl)			(47.4)		(44.6)		(43.5)	
Total cholesterol	26889	2199	203.1	6617	210.3	18073	206.2	<0.001
(mg/dl)			(61.2)		(60.8)		(59.9)	
Triglycerides	25629	2107	175.5	6322	223.0	17200	241.9	<0.001
(mg/dl)			(139.4)		(155.0)		(162.2)	
HDL cholesterol	14869	1194	47.5	4156	42.7	9519	40.1	<0.001
in men (mg/dl)			(21.3)		(17.1)		(16.9)	
HDL cholesterol	9872	809	57.3	1843	51.1	7220	46.3	<0.001
in women (mg/dl)			(26.8)		(23.4)		(18.7)	

LDL cholesterol, low-density lipoprotein cholesterol; HDL cholesterol, high-density lipoprotein cholesterol. Data presented as mean ±SD or percentage (n/N). P-values calculated using chi-squared test or Kruskal-Wallis-test.

### Antihyperglycemic therapy and comedication

Main antidiabetic drugs in participants with BMI <25 kg/m^2^ were metformin and insulin. While the percentage of metformin therapy increased significantly with increasing BMI (27.1% vs. 43.5% and 52.0%; P<0.001), more participants with BMI <25 kg/m^2^ were treated with short and long acting insulin in comparison to the other subgroups (short acting insulin 33.1% vs. 28.4% and 28.0%; P<0.001; long acting insulin 31.3% vs. 28.9% and 29.3%; P = 0.043) (Table 3). Sulfonylurea (SU) use ranged between 7.6 to 7.8%, with no significant difference between all subgroups. New antihyperglycemic drugs as DPP-IV inhibitors and GLP-1 analogues were used significantly more often in the BMI subgroups ≥ 25 kg/m^2^. The use of antihypertensives, lipid lowering drugs and platelet inhibitors was significantly lower in the lean BMI subgroup.

**Table 3:** Antidiabetic, antihypertensive and lipid lowering therapy.

	BMI	BMI	BMI	P-value
	<25 kg/m^2^	≥25-<30 kg/m^2^	≥30 kg/m^2^	
	N = 3,191	N = 9,234	N = 25,445	
**Antidiabetic drugs**				
Metformin (%)	27.1	43.5	52.0	<0.001
SU (%)	7.6	8.0	7.8	0.705
Glucosidase inhibitors (%)	0.8	0.7	0.9	0.320
Glinides (%)	3.4	3.3	2.6	<0.001
Glitazones (%)	0.7	1.5	1.9	<0.001
DPP-IV inhibitors (%)	7.8	10.2	11.3	<0.001
GLP-1 analogues (%)	0.8	2.2	8.1	<0.001
Short acting insulin (%)	33.1	28.4	28.0	<0.001
Long acting insulin (%)	31.3	28.9	29.3	0.043
**Antihypertensive drugs**				
ARBs (%)	2.0	3.8	6.8	<0.001
Betablockers (%)	7.2	9.4	13.9	<0.001
CCBs (%)	2.6	3.7	7.6	<0.001
ACEi (%)	9.6	14.6	20.6	<0.001
Diuretics (%)	5.0	6.9	12.9	<0.001
Platelet inhibitors	4.0	4.6	5.5	<0.001
**LLT**				
Statins (%)	10.1	14.3	15.7	<0.001
Ezetimibe (%)	0.4	0.6	0.8	0.003
Fibrate (%)	0.6	1.3	1.4	<0.001
Nicotinic acid (%)	0.1	0.1	0.1	0.696
other (%)	1.0	1.5	1.8	<0.001

OR, odds ratio; CI, 95% confidence interval; SU, sulfonylurea; DPP-IV, dipeptidyl peptidase inhibitor IV; GLP, glucagon like peptide; ARB, angiotensin receptor blocker; CCB, calcium channel blocker; ACEi, angiotensin converting enzyme inhibitor; LLT, lipid lowering therapy. Other: in 1.5% of the cohort with lipid-lowering drugs the type of lipid-lowering agent used was unknown. Data presented percentage (n/N). P-values calculated using chi-squared test.

### Predictors for being a lean participant with type 2 diabetes

The adjusted multivariable logistic regression analysis (Table 1) showed that being male had an odds ratio (OR, (confidence interval; CI)) of 1.19 (1.10; 1.29) for belonging to the BMI subgroup <25 kg/m^2^. Current smokers and participants with alcohol consumption ≥ 20 g had OR (CI) of 1.42 (1.30; 1.56) and 1.93 (1.60; 2.33), respectively. Interestingly, macrovascular comorbidities and comorbidities known as cardiovascular risk factors showed lower odds ratios as predictors for belonging to the lean BMI subgroup. Acute coronary syndrome and myocardial infarction had an OR (CI) of 0.78 (0.62; 0.96), history of myocardial infarction had an OR of 0.75 (0.59; 0.97), hypertension 0.29 (0.27; 0.32) and dyslipidemia 0.41 (0.37; 0.46).

### Events during the most recent treatment year in patients with lean versus obese type 2 diabetes

Looking at events during the most recent treatment year, lean patients did not have higher risks of myocardial infarction (1.22 (0.78; 1.91), stroke 1.00 (0.47; 2.08) or amputation 1.36 (0.94; 1.97) (Table 4). Hypoglycemic events were more frequent in the BMI subgroup < 25kg/m^2^ (3.0% vs. 1.7% and 1.3%; P< 0.001). Regression models adjusted for age, sex and diabetes duration showed a 2.50 times higher risk of hypoglycemia for lean participants compared to those with BMI ≥ 30 kg/m^2^ (Table 4). After additional adjustment for insulin therapy this difference persisted (OR 2.42 95%-CI: 1.83; 3.20). Death in association with the lean BMI group showed an odds ratio (CI) of 2.52 (1.60; 4.20).

**Table 4:** Events during the most recent treatment year in patients with lean versus obese type 2 diabetes.

	BMI	BMI	BMI	unadjusted	adjusted
	<25 kg/m^2^	≥25-<30 kg/m^2^	≥30 kg/m^2^	BMI <25 vs. ≥30	BMI <25 vs. ≥30
	N = 3,191	N = 9,234	N = 25,445	OR(95%CI)	OR (95%CI)^a^
	%	%	%		
**Non-fatal events**					
MI (%)	0.7	0.9	0.6	1.22	1.17
				(0.78; 1.91)	(0.74; 1.83)
Stroke (%)	0.3	0.3	0.3	1.00	1.00
				(0.48; 2.08)	(0.47; 2.05)
Amputation (%)	1.0	0.8	0.8	1.36	1.35
				(0.94; 1.97)	(0.93; 1.96)
Hypoglycaemia (%)	3.0	1.7	1.3	2.43	2.50
				(1.86; 3.16)	(1.89; 3.29)
**Death (%)**	0.7	0.3	0.3	2.59	2.52
				(1.60; 4.20)	(1.54; 4.13)

OR, odds ratio; CI, confidence interval; BMI, body mass index; MI, myocardial infarction

^a^adjusted for age, sex and diabetes duration

## Discussion

### Characteristics of patient population

In our cohort the subgroup of lean diabetes (8.4%) is smaller compared to previous studies [1–5] while the DiaRegis cohort also reported a quite small percentage of 7.5% [5].The lower prevalence of lean diabetics in our cohort might be the consequence of excluding participants with autoantibodies and wasting diseases. There was a preponderance of male participants in this group as described in the Chicago cohort and in an NHS cohort [1, 17]. Similar to data from these studies, our lean participants had less cardiovascular risk factors (hypertension, hypercholesterolemia) and less frequent a history of macrovascular diseases but showed a higher prevalence of alcohol use, cigarette smoking and chronic kidney disease.

In the lean diabetes group were significantly more smokers than in the other body weight groups. This is in accordance with numerous cross-sectional studies, that indicate that body weight, or body mass index (BMI; in kg/m2), is lower in cigarette smokers than in nonsmokers [18–21] A recent Mendelian randomization analysis also suggested that smoking causes lower body mass index (BMI) [22]. Smoking’s effect on body weight could lead to weight loss by increasing the metabolic rate, decreasing metabolic efficiency, or decreasing caloric absorption (reduction in appetite), all of which are associated with tobacco use. [23–25] tobacco consumption is clustered with other risk behaviors known to favor weight gain (eg, poor diet and low physical activity). These factors could counterbalance and even overtake the slimming effect of smoking. Overall, despite some conflicting observations, smoking is probably conducive to visceral fat accumulation and insulin resistance, and it increases the risk of metabolic syndrome and type 2 diabetes [23].

Concerning alcohol consumption 12% of lean diabetes patients but only 6.6% of the obese patients reported drinking more than 20g alcohol per day. This is higher than reported by the Chicago study, where the authors discuss an underreporting of alcohol consumption. But in accordance to the Chicago study in our study alcohol consumption is reported twice-fold higher in lean compared to obese patients [1]. Pathophysiologically chronic alcohol consumption induces pancreatic beta cell dysfunction and apoptosis [26]. For a large number of Japanese men who have relatively low BMI, alcohol intake is an established risk factor for diabetes. Since Japanese might have β cell dysfunctions such as being unable to compensate for diminished insulin sensitivity, it was speculated that the increased insulin sensitivity produced by alcohol intake, which would have a positive effect on prevention of diabetes, might not overcome its adverse effects in Japanese which might not be the case in westerners [27]. Indeed, several cohorts ranging from 10,482 to 138,031 individuals have shown no correlation (or a small negative correlation) between alcohol intake and BMI in men, and a small negative association with BMI in nutrient intake and relative body weight among US adults [28–35]. Other studies have found that alcohol intake is positively correlated with BMI in men or in both sexes; however, an analysis of recent studies suggests that this may be due to differences in intake patterns [29, 36]. For instance, several studies in adults have found that the amount or intensity of drinking per drinking occasion is positively correlated with BMI, while the frequency of drinking is negatively correlated, suggesting that frequent light drinking might offer a protective effect patterns and bodymass index in never smokers [37–39]. Furthermore, several studies have found that only excessive or heavy drinking is correlated with increased measures of adiposity [40]. Wannamethee, Shaper& Whincup found that in men, drinking <20 drinks per week was not associated with higher BMI, waist circumference (WC) or waist-to-hip ratio (WHR) compared to non-drinkers [41].

### Laboratory values

Blood glucose control, measured by HbA1c and fasting blood glucose differed statistically but not clinically significant between the different weight groups.These findings are different to the findings described in the Chicago study group, were lean participants showed worse blood glucose control compared to obese. In the DIVE/DPV cohort, we looked not only for differences in metabolic control but also for differences in hypoglycemia, with lean participants reporting significantly more hypoglycemic events. The lean participants had significantly better lipid profile butwith mean LDL-cholesterol of 119.7 mg/dl t treatment targets of < 70 mg/dl as recommended by ESC/EAS were not achieved even in lean patients [42]. This is in accordance to data of a German/Austrian DPV-multicenter analysis of 29,325 patients with type 2 diabetes and MI, or stroke, where treatment goals even in secondary prevention were reached in only 56.2% (MI), and 42.2% (stroke) [16]. Triglyceride to HDL-cholesterol ratios were lower in lean participants compared to obese as a marker for lower hepatic insulin resistance [43–45].

### Antihyperglycemic therapy and comedication

Main antidiabetic drugs used in our study cohort were metformin and insulin. In lean participants with BMI <25 kg/m^2^, metformin was prescribed only in 27.1%, significantly less compared to the other subgroups. While initially metformin was recommended by some guidelines only for overweight or obese participants with type 2 diabetes, since 2005, metformin is now the recommended first-line treatment independent of body weight [46, 47]. Metformin does not induce weight gain or hypoglycemia, and is the only diabetic treatment found to have a long-term benefit in reducing cardiovascular risks and organ damage [47, 48]. Thus it is surprising that only 27.1% of lean participants in our study, and even in participants with BMI ≥ 30 kg/m^2^ only 52.2%, were treated with metformin though HbA1c targets of <7% were not reached. Percentage of metformin usage in our cohort is in discordance to data from primary care in the UK, where in 2013, metformin prescribing was noted in 83.6% of the participants (95% CI 83.4% to 83.8%) [49]. On the other hand, insulin was significantly more often used in lean participants compared to the other subgroups: short acting insulin 33.1% and long acting insulin 31.3%. In the UK study only 15.1% of patients were prescribed insulin as single or combination therapy [49]. In our study, antihypertensive drugs were used significantly less often in lean participants compared to the overweight and obese subgroups. In all subgroups blood pressure targets were achieved by most patients. Lipid lowering therapy, mainly statins, was introduced only in a small percentage of participants in the cohort (10.1 to 15.7%) though treatment goals were not reached in the majority of subjects.

### Predictors for being a lean participant with type 2 diabetes

The typical lean participant with type 2 diabetes in our analysis was male, normotensive, and a current smoker with increased alcohol intake. Unhealthy lifestyle habits as higher prevalence of smoking and alcohol consumption in diabetic patients may have contributed to adverse effects and lead to higher risk of all-cause mortality in our cohort as described in the literature [50, 51].

### Events during the most recent treatment year in patients with lean versus obese type-2 diabetes

Analysis of events during the most recent treatment year showed no significant differences for macrovascular complications between lean and obese subgroups. However, participants with lean body weight had an OR of 2.43 for hypoglycemias compared to participants of the overweight subgroup.

The association between lean body weight and risk of hypoglycemia is also described in the Italian FADOI-DIAMOND study. In this study of patients with type 2 diabetes with BMI ≤ 25 kg/m^2^ vs. >25 kg/m^2^ (OR 1.39 (1.00–1.93), as well as patients with advanced age (>75 years vs. ≤ 75 years), cognitive dysfunction, and nephropathy, hospitalized in internal medicine wards had higher risks of hypoglycemic events [52]. In a retrospective cohort of 31,970 patients admitted to the general wards of an academic centre during 2007, those with hypoglycemic events had also a lower BMI, were older, had more co-morbidities, and received more insulin or sulfonylureas. There were fewer patients in the hypoglycemia group receiving metformin [53]. In a recent analysis of the DPV-Wiss database the risk of severe hypoglycemia in 29 485 sulfonylurea-treated participants with T2DM was also higher in participants with lower BMI. Adjusted event rates stratified by diabetes treatment were higher if sulfonylurea were combined with insulin: 6.7 events/100 patient-years (sulfonylurea + insulin), 4.9 events/100 patient-years (sulfonylurea + insulin + other OAD), 3.1 events/100 patient-years (sulfonylurea + other OAD) and 3.8 (sulfonylurea only) [54]. Analyses of time trends of antihyperglycaemic therapy and glycaemic control in 149,720 adult subjects with T2DM between 2002 and 2014 in DPV registry from Germany and Austria showed an increase in insulin therapy, particular as BOT while non-pharmacological therapy decreased [55].While risk of hypoglycemia often increases with tightening of blood glucose goals, the global HAT study showed an association between increased rates of hypoglycemia and duration of insulin therapy but no significant association with HbA1c level in T2DM [56]. In our study, participants with lean body weight had not only a higher OR for hypoglycemia but also an OR of 2.59 (95%CI 1.60; 4.20) for death compared to participants of the overweight subgroup. This increased mortality of lean participants is in accordance to data from a meta-analysis including sixteen studies with 385,925 participants [57]. The association between hypoglycemic events and mortality is widely recognized. ACCORD, ADVANCE and VADT study clearly demonstrated that an episode of severe hypoglycemia was associated with an increased risk of subsequent mortality. In a cohort of 20,005 participants aged 50 years or older with type 2 diabetes from the UK General Practice Research Database from November 1986 to November 2008, mortality was three times higher in patients in either the conventional or intensive treatment groups who had severe hypoglycemia than in those who did not have severe hypoglycemia [58]. Of course, in post hoc analyses a causal relationship cannot be established with certainty. It is possible that the association between hypoglycemia and death may be merely an indicator for vulnerability for death from any cause [59–61]. Similar to our observations, a retrospective analysis of the ACCORD study showed a greater hazard ratio for death among participants in the standard treatment arm in whom hypoglycemia occurred despite a high hemoglobin A1C level. In such patients, some hypoglycemia would occur because of an inherent instability of glucose control, whereas other episodes would be a direct result of the treatment regimen [59]. In concordance with these findings, in our study OR for hypoglycemia was increased in lean participants even after adjustment for insulin therapy, which points towards a more instable glucose metabolism in these patients.

### Limitations and strengths

The main strength of our study was the large number of participants included which was possible by analyzing data from two big German registries. We chose the age group between 30 and 50 ages to exclude participants with juvenile diabetes and on the other hand, participants with low BMI due to frailty and sarcopenia in older age. It was possible to exclude participants with a history of chronic and wasting diseases, which are strong confounders of body weight and mortality from DPV but not from the DIVE database. Analyzing participants with diabetes in the age between 30 and 50 years with lean phenotype as in our study implicates the risk to include up to 20% of patients with LADA [62]. Fortunately, we were able to exclude participants with positive autoantibodies (2.163 from DPV and 11 from DIVE). Nevertheless, undiagnosed participants with LADA or other diabetes types clinically resembling T2DM with lean phenotype cannot completely be excluded from our study population.

### Clinical implications and conclusions

In our analysis, lean participants with type 2 diabetes had higher mortality and an increased risk of hypoglycemic events. Use of metformin is recommended in guidelines but reported only in a minor part of lean patients while treatment strategies often included insulin, which is associated with increased hypoglycemia. Higher prevalence of smoking and alcohol consumption in these participants may also contribute to adverse effects and lead to higher risk of all-cause mortality seen in our cohort. The most appropriate treatment target in lean T2DM is reduction in mortality and cardiovascular risk. Although smoking cessation, alcohol reduction, physical activity, metformin, statins and angiotensin converting enzyme inhibitors have shown their efficacy to reduce cardiovascular mortality. Treatment strategies for lean participants with T2DM should therefore be multimodal with lifestyle modification, antihypertensive therapy, lipid lowering therapy and antihyperglycemic therapy avoiding hypoglycemia and insulin therapy, where possible.
